# Brain GABA and Glutamate Concentrations Following Chronic Gabapentin Administration: A Convenience Sample Studied During Early Abstinence From Alcohol

**DOI:** 10.3389/fpsyt.2018.00078

**Published:** 2018-03-15

**Authors:** Dieter J. Meyerhoff, Donna E. Murray, Timothy C. Durazzo, David L. Pennington

**Affiliations:** ^1^Department of Radiology and Biomedical Imaging, VA Medical Center, University of California San Francisco, San Francisco, CA, United States; ^2^VA Palo Alto Health Care System, Department of Psychiatry and Behavioral Sciences, Stanford University, Palo Alto, CA, United States; ^3^Department of Psychiatry, VA Medical Center, University of California San Francisco, San Francisco, CA, United States

**Keywords:** gabapentin, alcohol use disorders, magnetic resonance spectroscopy, GABA, glutamate, alcohol dependence, abstinence or withdrawal

## Abstract

Gabapentin (GBP), a GABA analog that may also affect glutamate (Glu) production, can normalize GABA and Glu tone during early abstinence from alcohol, effectively treating withdrawal symptoms and facilitating recovery. Using *in vivo* magnetic resonance spectroscopy, we tested the degree to which daily GBP alters regional brain GABA and Glu levels in short-term abstinent alcohol-dependent individuals. Regional metabolite levels were compared between 13 recently abstinent alcohol-dependent individuals who had received daily GBP for at least 1 week (GBP+) and 25 matched alcohol-dependent individuals who had not received GBP (GBP−). Magnetic resonance spectra from up to five different brain regions were analyzed to yield absolute GABA and Glu concentrations. GABA and Glu concentrations in the parieto-occipital cortex were not different between GBP− and GBP+. Glu levels in anterior cingulate cortex, dorsolateral prefrontal cortex, and basal ganglia did not differ between GBP− and GBP+. However, in a subgroup of individuals matched on age, sex, and abstinence duration, GBP+ had markedly lower Glu in the frontal white matter (WM) than GBP−, comparable to concentrations found in light/non-drinking controls. Furthermore, lower frontal WM Glu in GBP+ correlated with a higher daily GBP dose. Daily GBP treatment at an average of 1,600 mg/day for at least 1 week was not associated with altered cortical GABA and Glu concentrations during short-term abstinence from alcohol, but with lower Glu in frontal WM. GBP for the treatment of alcohol dependence may work through reducing Glu in WM rather than increasing cortical GABA.

## Introduction

Orally administered gabapentin (GBP), a GABA analog and anticonvulsant that is easily absorbed and crosses the blood–brain barrier, has shown promise in treating seizures and neuropathic pain. It is currently approved to treat seizure disorders by decreasing seizure activity presumably *via* increasing GABAergic tone and counter-balancing glutamatergic excitability (1). Monotherapy with GBP is also used to manage alcohol withdrawal and dependence in recovering alcohol-dependent individuals [for review see Leung et al. (2)]; it has been shown to reduce alcohol craving and use as well as improve sleep, anxiety, and mood disturbances commonly present in early abstinence. Thus, GBP facilitates recovery in alcohol-dependent individuals with little to no risk for abuse potential and liver damage, all with good tolerability and safety profile [e.g., Ref. (3, 4)]. Mechanistically, GBP is thought to normalize GABAergic tone *via* stimulating GABA synthesis and to affect glutamate (Glu) production in both neurons and glia (3, 5, 6), thereby affecting the excitability of postsynaptic membranes.

*In vivo* proton magnetic resonance spectroscopy (1H MRS) can quantify both GABA and Glu concentrations in the brain. When given orally to healthy human volunteers, a single dose of 17 mg/kg (7) or 900 mg of GBP (8) moderately increased MRS-detected GABA levels in the visual cortex within a few hours, while leaving occipital cortical Glu levels unchanged (8); the lower the individual’s basal occipital GABA level, the greater the measured GABA increase to the fixed GBP dose. By contrast, single doses of 150 or 300 mg of GBP did not change MRS-detectable GABA or Glu levels in the prefrontal or occipital cortices of healthy controls (9). In epileptic patients, 1H MRS demonstrated dose-dependent increases of initially low occipital cortical GABA levels after an initial dose and—to a lesser degree—after daily treatment with GBP (10–12). As with acute GBP administration to healthy controls (8), epilepsy patients with the greatest GABA increases had the lowest pretreatment GABA levels. Furthermore, the epilepsy patients who demonstrated the best seizure control with GBP administration had above normal brain GABA concentrations. Glu levels were not assessed in these epilepsy studies.

For many years, MRS has been employed to measure GABA, Glu, and other metabolites throughout the brain of alcohol-dependent individuals in outpatient treatment (ALC) who are not in acute withdrawal [for review see Meyerhoff (13)]. We observed that cortical GABA levels measured at approximately 1 week and 1 month of abstinence from alcohol did not differ significantly from those in non/light-drinking controls (CON) (14–16). We also found that levels of cortical Glu and *N*-acetylaspartate (NAA, a biomarker of neuronal viability) were significantly lower in 1-week-abstinent ALC than CON, suggesting neuronal injury and glutamatergic dysfunction reflected in imbalances of the metabolic Glu pool (14). Cortical GABA levels did not change in these ALC individuals between 1 and 5 weeks of abstinence from alcohol, whereas cortical Glu and NAA levels normalized over the same abstinence interval (14). During acute withdrawal, i.e., within a few days of sobriety, MRS-derived cortical Glu levels were elevated (17); however, we are not aware of any MRS studies that measured GABA levels during this time period. In frontal white matter (WM) and basal ganglia, our studies indicated lower NAA and myo-Inositol (mI, an astrocyte marker) in 4-week-abstinent ALC (18, 19) and additional strong trends to lower Glu and creatine-containing metabolites (Cr, including creatine and phosphocreatine, a high-energy metabolite) in basal ganglia (19); we did not measure GABA in these non-cortical brain regions. Taken together, these studies in short-term abstinent ALC suggest neuronal, glutamatergic and astrocytic abnormalities in fronto-striatal brain regions. Furthermore, cigarette smoking was shown associated with exacerbated reductions of regional NAA, Cr, and choline-containing metabolites (Cho) in ALC (20–22), with reduced dorsolateral prefrontal Glu concentration in non-alcoholic controls (23), and with NAA concentration deficits in the left hippocampus but not the anterior cingulate cortex (ACC) of smoking vs. non-smoking controls (24).

None of the ALC patients we previously studied (described above) had received GBP during their outpatient treatment, which typically consisted of stabilization/early recovery treatment for up to 30 days with psychosocial and cognitive behavioral programs attended 3–5 days per week. More recently, however, our referring treatment centers started managing their patients with GBP during early alcohol abstinence. This raised the question whether GABA, Glu, or the other commonly measured MRS metabolite levels in the cortex of ALC treated with daily GBP during outpatient treatment differed from the corresponding metabolite levels in ALC we previously investigated that were not treated with GBP. Therefore, we decided to formally compare these two recovering ALC groups on their brain metabolite levels in the various brain regions we have been typically examining in our addiction MRS research. We hypothesized higher GABA and similar Glu levels in cortical brain regions of patients chronically treated with GBP compared to those not treated with GBP. To further explore GBP’s potential tissue type-specific metabolic response and to help illuminate its mechanism of action, we also compared metabolite concentrations from subcortical brain regions and frontal WM.

## Materials and Methods

### Participants

Thirty-eight ALC (including eight females) between the ages of 25 and 60 years were referred by the VA Medical Center Substance Abuse Day Hospital and the Kaiser Permanente Chemical Dependence Recovery Program in San Francisco. In this naturalistic study design, 13 of these ALC had received daily oral GBP for at least 1 week before study (GBP+), whereas 25 ALC had not received GBP (GBP−) from their treatment provider at any time before study. The duration of GBP treatment before study and the daily dose were estimated from information obtained from treatment providers and corroborated by patient self-report. Given the nature of the data obtained and the fact that this was not a prospective treatment trial, only the daily dose data allowed some quantitative analyses; the GBP duration data were sufficiently incomplete for most patients to allow computing reliable quantitative estimates beyond the statement that all patients received GBP for at least 1 full week before study. Sixteen CON similar in age (48.7 ± 12.3 years) to our ALC group were recruited from the local community and studied contemporaneously with the ALC participants.

All study participants provided written informed consent prior to study per the Declaration of Helsinki and underwent procedures approved by the Institutional Review Board of the University of California San Francisco and the San Francisco VA Medical Center. Primary inclusion criteria for ALC participants were fluency in English, DSM-IV diagnosis of alcohol dependence or abuse at the time of enrollment, consumption of >150 standard alcohol-containing drinks (i.e., 13.6 g of ethanol/drink) per month for at least 8 years prior to enrollment for men, or consumption of >80 drinks/month for at least 6 years prior to enrollment for women. Exclusion criteria for ALC participants were history of the following: dependence on any substance other than alcohol or nicotine in the 5 years prior to enrollment, any intravenous drug use in the 5 years prior to study, current opioid agonist therapy, intrinsic cerebral masses or vascular malformations, HIV/AIDS, cerebrovascular accident, myocardial infarction, uncontrolled chronic hypertension, type-1 diabetes or insulin dependent conditions, moderate or severe COPD, non-alcohol related seizures, significant exposure to known neurotoxins, demyelinating and neurodegenerative diseases, documented Wernicke–Korsakoff syndrome, alcohol-induced persisting dementia, any penetrating head trauma, and closed head injury with loss of consciousness >10 min. Exclusion criteria also included history of schizophrenia-spectrum and bipolar disorders, PTSD, obsessive-compulsive disorder, and panic disorder. Hepatitis C, type-2 diabetes, hypertension, and unipolar mood disorder (major depression and/or substance-induced mood disorder) were permitted in the ALC cohort given their high prevalence in alcohol use disorders (AUDs) (25, 26). For ALC, inclusion/exclusion criteria were verified *via* electronic medical records, when available.

The ALC participants were involved in outpatient treatment at the time of assessment and none tested positive for alcohol or illicit substances (cannabinoids, cocaine, amphetamines, opioids, or PCP) before any of the assessments. Participants could be scanned only after their Clinical Institute Withdrawal Assessment for Alcohol Revised (CIWA-Ar) scores were below 10 (mild alcohol withdrawal is defined by a CIWA-Ar score less than or equal to 15). GBP− and GBP+ groups had a similar average duration of abstinence when studied. All CON participants were fluent in English, had no history of any DSM-IV Axis I disorder or biomedical condition that may have adversely affected their neurobiology, and they had not endorsed averaging >50 standard alcohol-containing drinks during any month over lifetime.

### Clinical and Neuropsychological Assessments

All ALC participants completed the Structured Clinical Interview for DSM-IV Axis I Disorders, Version 2.0 and CON were administered the accompanying screening module. Semi-structured interviews for lifetime alcohol consumption [Lifetime Drinking History (LDH)] (27, 28) and substance use (in-house questionnaire assessing substance type and quantity and frequency of use) (29) were administered to all participants. From the LDH, average number of alcoholic drinks per month over 1 year prior to enrollment, average number of drinks per month over lifetime, duration of heavy drinking (here operationalized as drinking >100 drinks per month in males, >80 drinks per month in females) were calculated. Premorbid verbal intelligence was estimated with the American National Adult Reading Test. Participants also completed standardized questionnaires assessing depressive (Beck Depression Inventory) (30) and anxiety symptoms (State-Trait Anxiety Inventory) (31), self-reported impulsivity (Barratt Impulsivity Scale) (32), and nicotine dependence *via* the Fagerstrom Tolerance Test for Nicotine Dependence (33). Table 1 shows demographics, behavioral measures, alcohol, and cigarette consumption variables for all GBP+ and GBP− participants. A standard neurocognitive battery was administered, test specific *Z*-scores were calculated based on corresponding normative data, cognitive domains were formed from specific neurocognitive tasks as previously described, and summary scores were calculated for each cognitive domain and global cognition (34).

**Table 1:** Demographic, clinical, and neuropsychological group characteristics.

Measure	GBP−	GBP+
*N* (including females)	25 (6)	13 (2)
Age (years)	47.4 ± 9.1	43.8 ± 11.5
Duration of abstinence (days)	19.3 ± 11.3	15.3 ± 10.4
AMNART	112 ± 9	117 ± 8
Beck Depression Inventory	13.0 ± 8.5	14.4 ± 8.4
Anxiety Inventory (State)	39.8 ± 13.7	33.4 ± 7.5
Anxiety Inventory (Trait)	45.9 ± 10.5	44.1 ± 14.5
Barratt Impulsivity Scale (total score)	64.6 ± 10.9	68.3 ± 10.6
Global cognition (*Z*-score)	−0.12 ± 0.54	−0.04 ± 0.74
Onset age of heavy drinking (years)	26.5 ± 9.6	26.0 ± 10.4
Duration of heavy drinking (months)	210 ± 129	191 ± 134
Drinks per month over lifetime	187 ± 130	177 ± 122
Drinks per month year before study	319 ± 230	258 ± 171
Duration of smoking (years)	23.4 ± 13.6	21.0 ± 11.1
Cigarette pack years	15.4 ± 13.3	9.3 ± 10.9
Fagerstrom total score*	3.9 ± 1.6 (*n* = 17)	2.6 ± 1.1 (*n* = 7)

*Mean ± SD. Only the Fagerstrom total score differed significantly between the smokers of the two groups (*p* = 0.05)*.

*p = 0.05.

*AMNART, American National Adult Reading Test; GBP, Gabapentin*.

### Imaging and Processing Method

**Figure 1:**
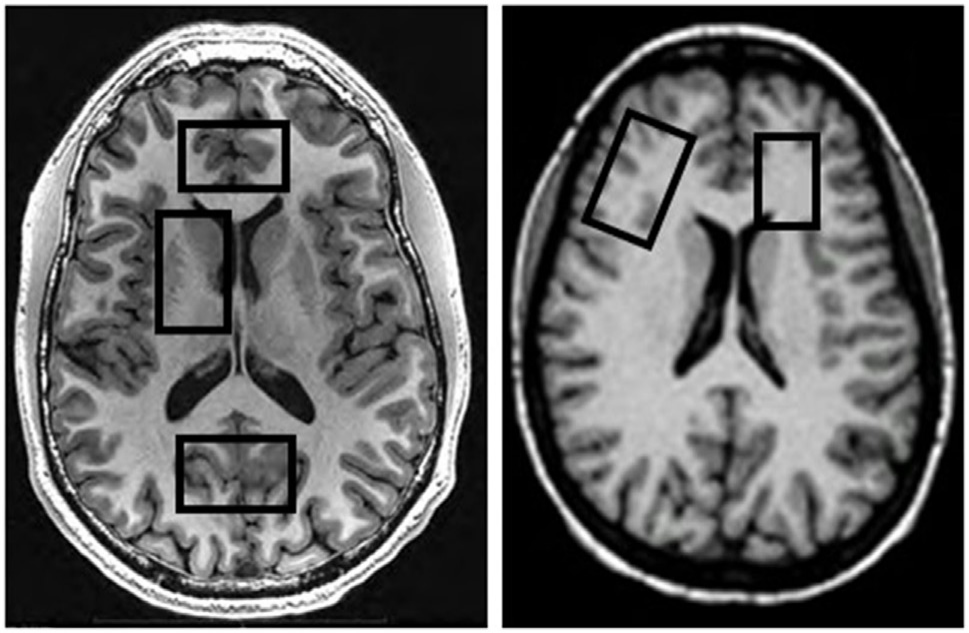
Typical locations of MRS volumes-of-interest on transverse MRIs.

MRI data were acquired on a 4 T MedSpec system using an 8-channel transmit-receive head coil and a Siemens Trio console (Siemens, Erlangen, Germany). A magnetization prepared rapid gradient (TR/TE/TI = 2,300/3/950 ms, 7° flip angle, 1 mm × 1 mm × 1 mm resolution) and a turbo spin-echo (TR/TE = 8,400/70 ms, 150° flip angle, 0.9 mm × 0.9 mm × 3 mm resolution) sequence were used to acquire 3-D sagittal T1-weighted and 2-D axial T2-weighted anatomical images, respectively. The images were then displayed on the scanner console for the prescription of up to five different MRS volumes-of-interest (VOIs) acquired in two scanning sessions (for typical VOI locations see Figure 1). After VOI-specific 3-D shimming and optimization of water suppression, J-edited GABA spectra were acquired from the POC (size: 40 mm × 20 mm × 20 mm) using a modified MEGA PRESS sequence (TR/TE = 2,000/71, editing frequencies at 1.9 and 7.5 ppm, 12.5 min acquisition time) (35), followed by the acquisition of a water spectrum from the same VOI. This was then followed by the acquisition of a stimulated echo acquisition mode (STEAM) sequence (TR/TE/TM = 2,000/12/10 ms, 2.5 min acquisition time) from the same VOI location. Additional STEAM metabolite and water spectra were acquired from the ACC (35 mm × 25 mm × 20 mm) and the right DLPFC (40 mm × 20 mm × 20 mm), with VOIs placed to maximize the inclusion of as much gray matter (GM) tissue as possible. For this analysis, GABA spectra from the ACC and DLPFC were available from all GBP− patients but only from two GBP+ patients, so that potential GABA differences in these VOIs could not be analyzed statistically.

In a second scan of all GBP+ patients generally within two days of the first scan, we further acquired metabolite and water STEAM spectra from the right basal ganglia (40 mm × 25 mm × 20 mm, including the head of the caudate, lenticular nucleus, and anterior thalamus) and the left frontal WM (25 mm × 15 mm × 20 mm), placed to include as much frontal WM tissue as possible (see Figure 1). Metabolite concentrations from these two subcortical VOIs were compared to the corresponding spectra available from a representative subgroup of 15 GBP− patients with characteristics like those of the entire GBP− group; these subcortical spectra had been obtained in the previous year on the same scanner, with the same software version and with identical experimental parameters. The five brain VOIs were chosen because of our group’s longstanding interest in regional metabolite concentration changes during abstinence from alcohol [for review see Meyerhoff (13)].

All metabolite and water MRS data were processed using established routines of a combination of in-house Matlab programs and IDL (Research Systems, Inc., Boulder, CO, USA) with SITOOLS (14, 36), yielding peak areas for total NAA, Glu, total Cr, total Cho, mI, and water for all five VOIs and additional GABA signal from cortical VOIs. At 4 T magnetic field strength, the Glu signal can be measured separately from the glutamine signal, and in our implementation of the GABA editing method, the measured GABA signal has some contribution from co-edited macromolecules. The T1 images obtained at the time of MRS were segmented into GM, WM, and cerebrospinal fluid (CSF) (37) and yielded estimates of the tissue fraction and CSF contributions to each VOI; these measures did not differ between GBP− and GBP+ for any of the analyzed VOIs. The individual spectral and segmentation information was then merged to calculate absolute molar concentrations [in institutional units (i.u.), i.e., without making assumptions about metabolite relaxation times] for metabolites in all VOIs, with details of these procedures described earlier (14).

### Data Analyses and Statistics

Demographic and clinical variables were compared between GBP+ and GBP− groups with *t*-tests and Fisher’s exact test, where indicated. We previously reported extensively on metabolite group differences between ALC (here called GBP−) and CON (14–16); consistent with those reports, in this new cohort, both NAA (*p* < 0.04) and Cr (*p* < 0.09) were lower in all three cortical VOIs of the entire ALC cohort of this report vs. CON. In this report, we focus on simple pairwise comparisons of region-specific metabolite levels between GBP+ and GBP−. Any such significant differences were then further explored by pairwise comparisons to CON. Because of our specific *a priori* hypotheses for group comparisons (cortical GABA elevated, Glu unchanged), an uncorrected *p* < 0.05 was considered statistically significant. In exploratory analyses, Pearson correlations were calculated for associations of behavioral and GBP dose data with spectral outcome measures and corresponding partial correlations were computed where indicated.

## Results

### Group Characteristics and GBP Dose

The GBP+ group was not significantly different from the GBP− group on sex distribution, average age, predicted premorbid intelligence, depressive and anxiety symptoms, self-reported impulsivity, common measures of lifetime alcohol consumption, and duration of abstinence from alcohol at the time of MR scan. The groups also did not differ significantly on cognitive domain summary scores (executive function, cognitive efficiency, processing speed, working memory, audioverbal learning and memory, visuospatial learning and memory, visuospatial skills, fine motor skills; all *p* > 0.29) or global cognition (see Table 1). The proportion of cigarette smokers was approximately equal between the GBP− (68%) and GBP+ groups (54%) and cigarette pack years as well as duration of lifetime smoking did not differ between the groups (all *p* > 0.25). However, the GBP+ had a lower Fagerstrom score than the GBP− group (*p* = 0.05), indicating low vs. moderate nicotine dependence, respectively.

At the time of the MR scan, GBP+ patients had received at least 1 week of oral GBP in three divided doses each day at a median daily dose of 1,200 mg (range: 800–3,600 mg, with 85% receiving between 800 and 1,800 mg; mean ± SD: 1,600 ± 900 mg). The large range of doses reflects different practices across treatment centers, different individual tolerance to the medication, and generally missing guidelines for the pharmacological treatment of alcohol dependence during early abstinence.

### Cortical Metabolite Measures

We compared cortical data from 13 GBP+ and 25 GBP− patients (unless otherwise specified). In the POC, none of the measured absolute metabolite concentrations (GABA, Glu, NAA, Cr, Cho, and mI) differed between GBP+ and GBP− (all *p* > 0.30). In the ACC, Glu, NAA, Cr, and Cho were equivalent between GBP+ and GBP− (all *p* > 0.20), while mI tended to be lower in GBP+ (*p* = 0.08). In the DLPFC, Glu, NAA, Cr, Cho, and mI were not different between GBP+ and GBP− (all *p* > 0.32). GABA concentrations from the ACC and DLPFC were only obtained in two GBP+ participants each and their values were within the range of the values obtained in the 25 GBP− patients.

### Subcortical Metabolite Measures

In the basal ganglia, similar to our findings in the ACC, the Glu, NAA, Cr, and Cho concentrations did not differ between 13 GBP+ and 15 GBP− patients (all *p* > 0.30), while mI tended to be lower in GBP+ (*p* = 0.08).

In the frontal WM, Glu was 15% lower in the 13 GBP+ vs. 15 GBP− patients (2.23 ± 0.39 vs. 2.61 ± 0.36, *p* = 0.017; Cohen’s *d* effect size = 0.99), while NAA, Cr, Cho, and mI were statistically equivalent between the groups (all *p* > 0.12). The lower frontal WM Glu in GBP+ remained significant after covarying for Fagerstrom total score, indicating that the Glu difference was independent of the severity of nicotine dependence. The lower frontal WM Glu of the GBP+ group showed a moderately strong, but non-significant correlation, with higher daily GBP dose (univariate *r* = −0.47, *p* = 0.12; partial *r* = −0.58, *p* = 0.13, with abstinence duration as covariate); by contrast, none of the cortical GABA and Glu concentrations correlated even weakly with GBP dose. The frontal WM Glu in GBP+ was not associated with any measure of lifetime alcohol consumption, smoking severity, or age.

In group comparisons of basal ganglia and frontal WM metabolite levels available from a subset of 10 of the 16 CON participants, frontal WM Glu was significantly higher in GBP− vs. CON (2.61 ± 0.36 vs. 2.20 ± 0.46, *p* = 0.026), whereas it was statistically equivalent between GBP+ and CON (2.23 ± 0.39 vs. 2.20 ± 0.46, *p* = 0.85). The combined group of ALC participants showed no such Glu difference compared to the subset of CON (*p* > 0.18). Furthermore, the GBP+ and GBP− groups did not differ from CON on any of the other regional metabolite concentrations.

**Figure 2:**
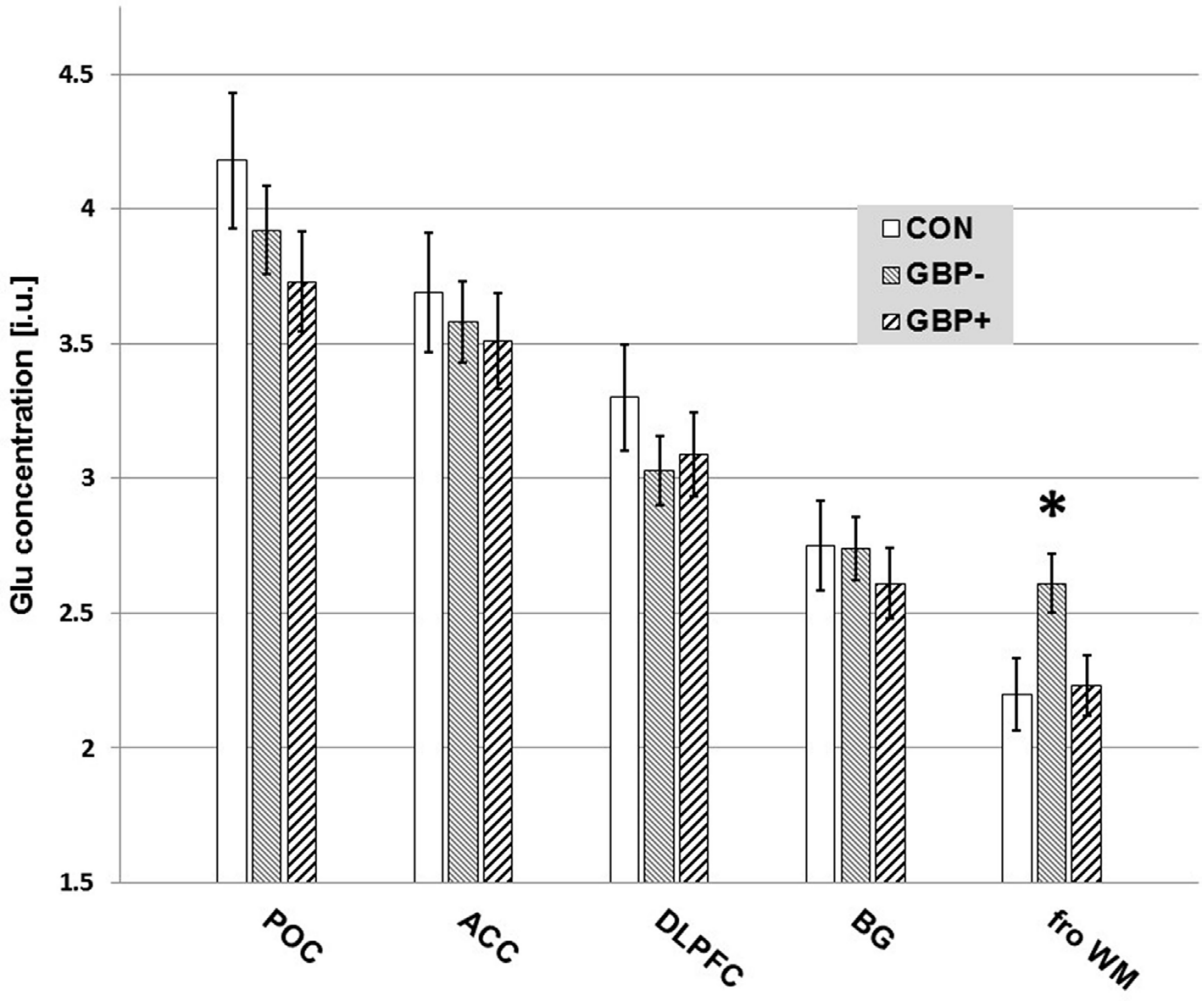
Measured glutamate (Glu) concentrations [institutional units (i.u.)] by volume-of-interest and group. Mean ± SE. Only the differences in frontal white matter (fro WM) Glu of GBP− to GBP+ (*p* = 0.017) and controls (CON) (*p* = 0.026) reached statistical significance.

All findings remained essentially the same when re-analyzing the data without the small number of women included in this U.S. Veterans sample. Figure 2 is a bar graph of Glu concentrations (i.u.) observed in the different VOIs across CON, GBP−, and GBP+ groups.

### Associations of Frontal WM Glu With Neuropsychological Measures

Within the ALC group (GBP+ and GBP− combined), all correlations of frontal WM Glu with the summary scores for cognitive domains and global cognition were positive (all *r* = 0.044–0.525), indicating that higher frontal WM Glu was associated with better cognitive function. This relationship was observed for both groups; also, recall that the GBP groups did not differ significantly on cognitive performance. The strongest and most significant correlations were observed with visuospatial memory (*r* = 0.525, *p* = 0.004) and visuospatial learning (*r* = 0.456, *p* = 0.015), while the corresponding correlations with cognitive efficiency and global cognition were at the trend level (*r* > 0.380, *p* < 0.075). None of the associations of frontal WM Glu with measures of self-reported impulsivity and anxiety was significant.

## Discussion

We tested whether ALC in early recovery treated daily with oral GBP for at least 1 week had higher cortical GABA and altered tissue-specific Glu concentrations compared to demographically and clinically similar ALC not treated with GBP. Counter to the general notion and our hypothesis that GBP administration would elevate brain GABA levels, GABA concentration in the POC did not statistically differ between GBP− and GBP+ groups. In addition, Glu levels in POC and in our frontal cortical (ACC, DLPFC) and basal ganglia VOIs did not differ between the groups. As such, GBP treatment at a median dose of 1,200 mg/day for at least 1 week was not associated with altered cortical GABA or cortical and basal ganglia Glu levels in short-term abstinent ALC. However, ALC receiving GBP had markedly lower Glu in the frontal WM than matched ALC not receiving GBP, and this Glu reduction showed a moderately strong but not significant relationship with a higher estimated daily GBP dose. Furthermore, whereas the frontal WM Glu concentration was significantly elevated in GBP− relative to CON, it was normal in GBP+. Although we did not gather serial withdrawal- or craving-related data, behavioral or reliable subsequent relapse data, we tentatively conclude that GBP for the treatment of alcohol dependence and/or associated craving, insomnia, anxiety, or mood does not work primarily through increasing cortical GABA levels. Rather, chronic GBP treatment may work through reducing metabolic Glu in WM as previously suggested in general (3, 5, 6) and specifically for the treatment of alcohol withdrawal (3).

Notably, chronic GBP treatment in recovering ALC was not associated with elevated MRS-observable central GABA levels. Equivalent GABA levels in those treated and not treated with GBP and their normal levels compared to healthy controls [Ref. (14–16) and this study] contrast with conditions in epilepsy, where daily GBP treatment increases initially low GABA levels and reduces seizure activity (10, 12, 38). In further contrast to epilepsy studies, we also did not find a dependence of cortical GABA levels during treatment on GBP dose. Since we studied our patients 2–3 weeks after their last alcoholic drink when all potential acute withdrawal symptoms had subsided (CIWA-Ar score below 10), it is possible that potentially low GABA levels during acute withdrawal (and potentially higher CIWA-Ar scores) had already normalized in these patients. In that phase, continued treatment with GBP may not further increase brain GABA levels. This explanation is reminiscent of studies performed in epilepsy patients, who showed a blunted GABA increase with daily GBP treatment after initially low GABA levels had normalized (12).

Also, cortical and basal ganglia Glu levels in our recovering ALC were not associated with chronic daily GBP treatment, consistent with the absence of occipital cortical Glu changes after oral GBP challenge in healthy controls (8, 9). To further explore GBP’s potential brain tissue-type specific metabolic sequelae, we also compared 1H MRS metabolic information from a prefrontal VOI that contained primarily WM in ALC receiving and not receiving daily GBP and found significantly lower frontal WM Glu in GBP+. The observed WM Glu level in our human studies was reduced as a function of the administered daily GBP dose, but not as a function of prior alcohol consumption. These tissue-specific Glu alterations suggest that daily GBP administration in early abstinence affects primarily and specifically cells in the WM and that those metabolic alterations may reflect the potential mechanisms of action of GBP, as previously suggested (3). Of note, GBP treatment normalized frontal WM Glu levels (i.e., it did not reduce frontal WM Glu below levels measured in CON). This may be clinically important, given that lower Glu in frontal WM was found associated with loss of control of alcohol use and severity of alcohol dependence (39).

These results of our human study are somewhat consistent with an *ex vivo* mouse study, in which chronic treatment with GBP at doses commensurate with those used in our patients did not affect global brain GABA levels but did reduce global Glu levels (40). The authors concluded that the clinical activity of GBP is not likely related to metabolic GABA or Glu levels, but potentially to neurotransmitter GABA levels that may have only increased in discrete brain regions.

Our study is distinctive in the number of brain regions investigated spectroscopically in any alcohol-dependent cohort and in addressing the question of potential metabolic effects of daily oral GBP in recovering ALC. Whereas the findings contribute to our understanding of GBP’s potential neurobiological mechanism of action, they also have limitations: one, the cohort constituted a modestly sized convenience sample of treatment-seeking individuals with AUDs. Two, while both men and women were studied (with comparable proportions across the groups), the women were not studied at the same phase of their menstrual cycle. As GABA levels vary as a function of the menstrual phase (41), corresponding noise in the data may have reduced our ability to detect GABA group differences. However, our findings remained essentially the same when re-analyzing the data without the women included. Three, our study was observational and participants were not randomly assigned to the two study groups; furthermore, due to different abilities to endure long scan times, not all participants contributed spectral data from all five VOIs. Therefore, salient differences may exist between the groups analyzed that we had not considered. This limitation is somewhat mitigated, however, by our main comparison groups not differing on many potentially confounding clinical, demographic, and behavioral variables. Nevertheless, it is still possible that daily GBP treatment affects selectively discrete and functionally relevant neurotransmitter pools of GABA, which cannot be separately observed by our method. Finally, we report on cross-sectional comparisons between carefully matched samples studied after acute withdrawal; only serial MRS bracketing the acute withdrawal phase with data obtained before and after a prospective and well-controlled daily GBP administration protocol will definitively link causation to symptom and metabolite level changes.

In summary, in treatment-seeking ALC at 2–3 weeks of abstinence, daily administration of GBP at an average dose of 1,600 mg/day for at least 1 week before study was not associated with altered 1H MRS-observable cortical GABA, Glu, or other metabolite levels. As such, in MRS studies of cortical metabolite concentrations during abstinence from alcohol, patients on this GBP regimen (or on lower doses) can be pooled for data analyses with patients not on GBP. However, daily GBP treatment was associated with uniquely lower frontal Glu in a predominantly WM VOI, to the degree that Glu levels were equivalent to those in non/light-drinking healthy controls. Rather than pointing to GBP’s ability to increase the metabolic pool of cortical GABA during early abstinence from alcohol (*via* promoting GABA synthesis and/or boosting GABA levels), the observed metabolic effects may point to primary WM involvement—glial and/or axonal—in the mechanism of action of GBP during early abstinence, potentially *via* activating glutamate dehydrogenase (42). Thus, in regard to examining WM metabolite levels during abstinence from alcohol, patient groups on and off GBP should be analyzed separately.

## Ethics Statement

This study was carried out in accordance with the recommendations of and after approval from the Human Research Protection Program Institutional Review Board (IRB) of the University of California San Francisco and the San Francisco VA Medical Center. All study participants provided written informed consent in accordance with the Declaration of Helsinki before any study procedures.

## Author Contributions

DJM obtained funding for this study, conceptualized its design, processed spectral data, performed most of the statistical data analyses, interpreted the data, and wrote the manuscript. TD and DP recruited study participants and conducted their clinical and neuropsychological assessments. DEM performed spectral data processing and quantitation under DJM’s supervision. All authors contributed substantially to the content of the manuscript, critically reviewed the first draft, and approved the final version for publication.

## Conflict of Interest Statement

The authors declare that the research was conducted in the absence of any commercial or financial relationships that could be construed as a potential conflict of interest.

## References

[1] During MJ Spencer DD. Extracellular hippocampal glutamate and spontaneous seizure in the conscious human brain. Lancet (1993) 341:1607–10. 10.1016/0140-6736(93)90754-5 8099987

[2] Leung JG Hall-Flavin D Nelson S Schmidt KA Schak KM. The role of gabapentin in the management of alcohol withdrawal and dependence. Ann Pharmacother (2015) 49:897–906. 10.1177/1060028015585849 25969570

[3] Bonnet U Banger M Leweke FM Maschke M Kowalski T Gastpar M. Treatment of alcohol withdrawal syndrome with gabapentin. Pharmacopsychiatry (1999) 32:107–9. 10.1055/s-2007-979203 10463378

[4] Schacht JP Randall PK Waid LR Baros AM Latham PK Wright TM Neurocognitive performance, alcohol withdrawal, and effects of a combination of flumazenil and gabapentin in alcohol dependence. Alcohol Clin Exp Res (2011) 35:2030–8. 10.1111/j.1530-0277.2011.01554.x 21631542 PMC3166540

[5] Gotz E Feuerstein TJ Lais A Meyer DK. Effects of gabapentin on release of gamma-aminobutyric acid from slices of rat neostriatum. Arzneimittelforschung (1993) 43:636–8. 8394711

[6] Sills GJ. The mechanisms of action of gabapentin and pregabalin. Curr Opin Pharmacol (2006) 6:108–13. 10.1016/j.coph.2005.11.003 16376147

[7] Kuzniecky R Ho S Pan J Martin R Gilliam F Faught E Modulation of cerebral GABA by topiramate, lamotrigine, and gabapentin in healthy adults. Neurology (2002) 58:368–72. 10.1212/WNL.58.3.368 11839834

[8] Cai K Nanga RPR Lamprou L Schinstine C Elliott M Hariharan H The impact of gabapentin administration on brain GABA and glutamate concentrations: a 7T (1)H-MRS study. Neuropsychopharmacology (2012) 37:2764–71. 10.1038/npp.2012.142 22871916 PMC3499716

[9] Preuss N Van Der Veen JW Carlson PJ Shen J Hasler G. Low single dose gabapentin does not affect prefrontal and occipital gamma-aminobutyric acid concentrations. Eur Neuropsychopharmacol (2013) 23:1708–13. 10.1016/j.euroneuro.2013.08.006 24071367

[10] Petroff OA Rothman DL Behar KL Lamoureux D Mattson RH The effect of gabapentin on brain gamma-aminobutyric acid in patients with epilepsy. Ann Neurol (1996) 39:95–9. 10.1002/ana.410390114 8572673

[11] Petroff OA Rothman DL Behar KL Mattson RH Low brain GABA level is associated with poor seizure control. Ann Neurol (1996) 40:908–11. 10.1002/ana.410400613 9007096

[12] Petroff OA Hyder F Rothman DL Mattson RH. Effects of gabapentin on brain GABA, homocarnosine, and pyrrolidinone in epilepsy patients. Epilepsia (2000) 41:675–80. 10.1111/j.1528-1157.2000.tb00227.x 10840398

[13] Meyerhoff DJ. Brain proton magnetic resonance spectroscopy of alcohol use disorders. Handb Clin Neurol (2014) 125:313–37. 10.1016/B978-0-444-62619-6.00019-7 25307583 PMC4876042

[14] Mon A Durazzo TC Meyerhoff DJ. Glutamate, GABA, and other cortical metabolite concentrations during early abstinence from alcohol and their associations with neurocognitive changes. Drug Alcohol Depend (2012) 125:27–36. 10.1016/j.drugalcdep.2012.03.012 22503310 PMC3419314

[15] Abé C Mon A Durazzo TC Pennington DL Schmidt TP Meyerhoff DJ. Polysubstance and alcohol dependence: unique abnormalities of magnetic resonance-derived brain metabolite levels. Drug Alcohol Depend (2013) 130:30–7. 10.1016/j.drugalcdep.2012.10.004 23122599 PMC3624044

[16] Murray DE Durazzo TC Schmidt TP Abé C Guydish J Meyerhoff DJ. Frontal metabolite concentration deficits in opiate dependence relate to substance use, cognition, and self-regulation. J Addict Res Ther (2016) 7:286–96. 10.4172/2155-6105.1000286 27695638 PMC5042152

[17] Hermann D Weber-Fahr W Sartorius A Hoerst M Frischknecht U Tunc-Skarka N Translational magnetic resonance spectroscopy reveals excessive central glutamate levels during alcohol withdrawal in humans and rats. Biol Psychiatry (2012) 71:1015–21. 10.1016/j.biopsych.2011.07.034 21907974

[18] Wang JJ Durazzo TC Gazdzinski S Yeh PH Mon A Meyerhoff DJ. MRSI and DTI: a multimodal approach for improved detection of white matter abnormalities in alcohol and nicotine dependence. NMR Biomed (2009) 22:516–22. 10.1002/nbm.1363 19156697 PMC4156512

[19] Murray DE Durazzo TC Schmidt TP Abé C Mon A Guydish JR Altered proton metabolite levels in frontal and subcortical brain regions of opiate dependent individuals on buprenorphine maintenance. Alcohol Clin Exp Res (2015) 39:178A.

[20] Durazzo TC Gazdzinski S Banys P Meyerhoff DJ. Cigarette smoking exacerbates chronic alcohol-induced brain damage: a preliminary metabolite imaging study. Alcohol Clin Exp Res (2004) 28:1849–60. 10.1097/01.ALC.0000148112.92525.AC 15608601

[21] Durazzo TC Gazdzinski S Rothlind JC Banys P Meyerhoff DJ. Brain metabolite concentrations and neurocognition during short-term recovery from alcohol dependence: preliminary evidence of the effects of concurrent chronic cigarette smoking. Alcohol Clin Exp Res (2006) 30:539–51. 10.1111/j.1530-0277.2006.00060.x 16499496

[22] Durazzo TC Mon A Gazdzinski S Meyerhoff DJ. Chronic cigarette smoking in alcohol dependence: associations with cortical thickness and N-acetylaspartate levels in the extended brain reward system. Addict Biol (2013) 18:379–91. 10.1111/j.1369-1600.2011.00407.x 22070867 PMC4157587

[23] Durazzo TC Meyerhoff DJ Mon A Abe C Gazdzinski S Murray DE. Chronic cigarette smoking in healthy middle-aged individuals is associated with decreased regional brain N-acetylaspartate and glutamate levels. Biol Psychiatry (2016) 79:481–8. 10.1016/j.biopsych.2015.03.029 25979621 PMC4600002

[24] Gallinat J Lang UE Jacobsen LK Bajbouj M Kalus P Von Haebler D Abnormal hippocampal neurochemistry in smokers: evidence from proton magnetic resonance spectroscopy at 3 T. J Clin Psychopharmacol (2007) 27:80–4. 10.1097/JCP.0b013e31802dffde 17224719

[25] Mertens JR Lu YW Parthasarathy S Moore C Weisner CM. Medical and psychiatric conditions of alcohol and drug treatment patients in an HMO: comparison with matched controls. Arch Intern Med (2003) 163:2511–7. 10.1001/archinte.163.20.2511 14609789

[26] Stinson FS Grant BF Dawson DA Ruan WJ Huang B Saha T. Comorbidity between DSM-IV alcohol and specific drug use disorders in the United States: results from the National Epidemiologic Survey on Alcohol and Related Conditions. Drug Alcohol Depend (2005) 80:105–16. 10.1016/j.drugalcdep.2005.03.009 16157233

[27] Skinner HA Sheu WJ Reliability of alcohol use indices. The Lifetime Drinking History and the MAST. J Stud Alcohol (1982) 43:1157–70. 10.15288/jsa.1982.43.1157 7182675

[28] Sobell LC Sobell MB Riley DM Schuller R Pavan DS Cancilla A The reliability of alcohol abusers’ self-reports of drinking and life events that occurred in the distant past. J Stud Alcohol (1988) 49:225–32. 10.15288/jsa.1988.49.225 3374136

[29] Pennington DL Durazzo TC Schmidt TP Abe C Mon A Meyerhoff DJ. Alcohol use disorder with and without stimulant use: brain morphometry and its associations with cigarette smoking, cognition, and inhibitory control. PLoS One (2015) 10:e0122505. 10.1371/journal.pone.0122505 25803861 PMC4372577

[30] Beck AT Depression Inventory. Philadelphia: Center for Cognitive Therapy (1978).

[31] Spielberger CD Gorsuch RL Lushene R Vagg PR Jacobs GA Manual for the State-Trait Anxiety Inventory. Palo Alto, CA: Consulting Psychologists Press (1983).

[32] Patton JH Stanford MS Barratt ES. Factor structure of the Barratt Impulsiveness Scale. J Clin Psychol (1995) 51:768–74. 10.1002/1097-4679(199511)51:6<768::AID-JCLP2270510607>3.0.CO;2-1 8778124

[33] Fagerstrom KO Heatherton TF Kozlowski LT Nicotine addiction and its assessment. Ear Nose Throat J (1991) 69:763–5. 2276350

[34] Durazzo TC Rothlind JC Gazdzinski S Banys P Meyerhoff DJ. Chronic smoking is associated with differential neurocognitive recovery in abstinent alcoholic patients: a preliminary investigation. Alcohol Clin Exp Res (2007) 31:1114–27. 10.1111/j.1530-0277.2007.00398.x 17451399

[35] Kaiser LG Young K Meyerhoff DJ Mueller SG Matson GB. A detailed analysis of localized J-difference GABA editing: theoretical and experimental study at 4 T. NMR Biomed (2008) 21:22–32. 10.1002/nbm.1150 17377933

[36] Soher BJ Van Zijl PC Duyn JH Barker PB Quantitative proton MR spectroscopic imaging of the human brain. Magn Reson Med (1996) 35:356–63. 10.1002/mrm.1910350313 8699947

[37] Van Leemput K Maes F Vandermeulen D Suetens P. Automated model-based bias field correction of MR images of the brain. IEEE Trans Med Imaging (1999) 18:885–96. 10.1109/42.811268 10628948

[38] Petroff OA Behar KL Rothman DL New NMR measurements in epilepsy. Measuring brain GABA in patients with complex partial seizures. Adv Neurol (1999) 79:939–45. 10514877

[39] Ende G Hermann D Demirakca T Hoerst M Tunc-Skarka N Weber-Fahr W Loss of control of alcohol use and severity of alcohol dependence in non-treatment-seeking heavy drinkers are related to lower glutamate in frontal white matter. Alcohol Clin Exp Res (2013) 37:1643–9. 10.1111/acer.12149 23800328

[40] Leach JP Sills GJ Butler E Forrest G Thompson GG Brodie MJ. Neurochemical actions of gabapentin in mouse brain. Epilepsy Res (1997) 27:175–80. 10.1016/S0920-1211(97)01034-6 9237051

[41] Epperson CN Haga K Mason GF Sellers E Gueorguieva R Zhang W Cortical gamma-aminobutyric acid levels across the menstrual cycle in healthy women and those with premenstrual dysphoric disorder: a proton magnetic resonance spectroscopy study. Arch Gen Psychiatry (2002) 59:851–8. 10.1001/archpsyc.59.9.851 12215085

[42] Cho SW Cho EH Choi SY. Activation of two types of brain glutamate dehydrogenase isoproteins by gabapentin. FEBS Lett (1998) 426:196–200. 10.1016/S0014-5793(98)00335-4 9599007

